# A Phase I, open-label, dose escalation study of MGA271 in combination with ipilimumab in patients with B7-H3-expressing melanoma, squamous cell cancer of the head and neck or non-small cell lung cancer

**DOI:** 10.1186/2051-1426-3-S2-P176

**Published:** 2015-11-04

**Authors:** Walter Urba, Bartosz Chmielowski, Deryk Loo, Jan Baughman, Francine Chen, Paul Moore, Ezio Bonvini, James Vasselli, Jon Wigginton, Naiyer Rizvi

**Affiliations:** 1 Earle A. Chiles Research Institute, Portland, OR, USA; 2 Division of Hematology - Medical Oncology, UCLA Jonsson Comprehensive Cancer Center, Los Angeles, CA, USA; 3 MacroGenics, San Francisco, CA, USA; 4 MacroGenics, Rockville, MD, USA; 5 Columbia University Medical Center, New York, NY, USA

## Background

MGA271 is an Fc optimized, humanized IgG1 monoclonal antibody targeting B7-H3 (CD276), a member of the B7 family, and is currently undergoing Phase I testing. The Fc domain is engineered for enhanced binding to the activating FcγR, CD16A, and decreased binding to the inhibitory FcγR, CD32B. B7-H3 exhibits limited expression on normal tissues, but is highly expressed in a broad range of tumors, including melanoma (M), squamous cell cancer of the head and neck (SCCHN) and non-small cell lung cancer (NSCLC). The correlation between B7-H3 overexpression and poor prognosis in certain cancers suggests a role for B7-H3 in tumor immune escape. Although ipilimumab (anti-CTLA-4) is approved for the treatment of melanoma, only a minority of patients respond clinically. Combined targeting of CTLA-4 and PD-1, using ipilimumab and nivolumab (anti-PD-1), demonstrated synergistic antitumor activity, albeit with substantially increased toxicity over either agent alone. B7-H3 and CTLA-4 play distinct immunoregulatory roles and it appears that complementary mechanisms could be engaged by targeting these molecules. These include modulation of T-cell function, including the function of regulatory T-cells, and engagement of both innate and adaptive immunity. Further, B7-H3 expression in normal tissues is limited, suggesting that B7-H3 may not be a key driver in maintaining self-tolerance, thus reducing the potential for induction of immune-related AEs (irAEs) in patients treated with MGA271 compared to molecules such as anti-CTLA-4. Based on these observations, we hypothesized that MGA271 could potentiate the immunoregulatory and antitumor activity of ipilimumab, with a more favorable overall safety profile than other combinations such as anti-CTLA-4/anti-PD-1.

## Methods

This multi-center, open-label trial (NCT02381314) enrolls patients with advanced B7-H3-expressing SCCHN, M, or NSCLC (squamous and non-squamous histology). Progression on previous checkpoint inhibitor therapy is allowed. To investigate whether B7H3 contributes to “immunologic escape” in patients treated with checkpoint inhibitors, M patients are required to have progressed on a checkpoint inhibitor within 12 weeks of enrollment. Using a 3+3 +3 dose escalation design, successive cohorts of patients will be treated with escalating doses of weekly IV MGA271 (starting dose 3 mg/kg) up to 1 year in combination with standard doses of IV ipilimumab (3 mg/kg) Q3 weeks x 4 doses. Cohort expansions will be enrolled at the maximum tolerated dose, and include patients with M, HNSC and NSCLC (16 patients/cohort). Major objectives for this study include characterization of safety, tolerability, pharmacokinetics, pharmacodynamics, and preliminary antitumor activity of MGA271 in combination with ipilimumab.

## Trial registration

ClinicalTrials.gov identifier NCT02381314.

